# The effectiveness of a theory -based health education program on waterpipe smoking cessation in Iran: one year follow-up of a quasi-experimental research

**DOI:** 10.1186/s12889-024-18169-7

**Published:** 2024-03-01

**Authors:** Nahid Shahabi, Shirin Shahbazi Sighaldeh, Hadi Eshaghi Sani Kakhaki, Shokrollah Mohseni, Sara Dadipoor, Omar El-Shahawy

**Affiliations:** 1 Social Determinants in Health Promotion Research Center, Hormozgan Health Institute, Hormozgan University of Medical Sciences, Bandar Abbas, Iran; 2 Midwifery and Reproductive Health Department, School of Nursing and Midwifery, Tehran Universities of Medical Sciences, Tehran, Iran; 3 Nursing and Midwifery Care Research Center, Tehran University of Medical Sciences, Tehran, Iran; 4 Tobacco and Health Research Center, Hormozgan University of Medical Sciences, Bandar Abbas, Iran; 5 Department of Population Health, New York University Grossman School of Medicine, New York, NY USA

**Keywords:** Smoking, Tobacco, Waterpipe, Women, Prevention

## Abstract

**Background:**

The present research aimed to determine the effect of an educational intervention based on the extended theory of planned behavior (ETPB) on waterpipe (WT) smoking cessation in women.

**Methods:**

The present quasi-experimental had a pre-test, post-test design with 3, 6 and 12 months follow-ups was conducted in Bandar Abbas city, south of Iran in December 2021-March 2023. A total of 448 women over the age of 15 (224 in the intervention group (IG), 224 in the control group (CG)), using a two-stage cluster sampling method participated. The educational intervention focused on WT smoking cessation implemented in 14 sessions. The educational methods in the training sessions were lectures, collaborative discussions, Q&As, brainstorming, role plays, and peer education. The main outcome was WT cessation behavior. Repeated measures ANOVA tests and post hoc were run to compare the IG and CG at baseline in terms of demographic variables, t-test and chi square test, and in the four points of time of data collection. The data were analyzed in Stata14. A *p*-value < 0.05 was considered as statistically significant.

**Results:**

The mean and standard deviation of WT cessation behavior and all ETPB constructs in the IG was significantly higher than the CG. After the educational intervention, in the IG, the perceived behavioral control, attitude, subjective norm, intention and knowledge increased, and the weekly smoking and WT smoking habit decreased (*P* < 0.001). The CG did not have any significant change in other variables except for the increased knowledge score. During the 12-month follow-up, the cessation rate was 43.81% (*P* = 0.645) in the IG and 7.45% in the CG (*P* = 0.081).

**Conclusions:**

The educational intervention positively affected WT smoking reduction and cessation in women through influencing the ETPB constructs. It is strongly recommended to design theory-based interventions beyond the individual level with an emphasis on interpersonal relationships to facilitate WT cessation as far as possible.

**Supplementary Information:**

The online version contains supplementary material available at 10.1186/s12889-024-18169-7.

## Background

Waterpipe (WT) is one type of smoking material known in different countries by other names such as hookah, argileh, shisha, goza and narghile. WT is a device for consuming tobacco [1]. While it is a conventional method of tobacco consumption originating in the Middle East, it has become extremely popular on a global scale, especially among young adults and women [2, 3]. While the prevalence of WT is growing in many countries [4–7], Middle Eastern countries remains to lead these growing trends in WT use [8, 9]. A review reported the prevalence of WT smoking in the Eastern Mediterranean region ranged from 2.5% in Oman to 37.2% in Lebanon [10], and this rate was estimated at 10.2–11.3% in Iran [11]. The highest prevalence of WT smoking in Iran is in the southern region primarily among women [12, 13], where it was estimated in Hormozgan province to be at 10.3%, which is significantly higher than other provinces of Iran [13].

Contrary to the common belief, the continuous use of WT compared to cigarettes has more adverse effects on health [14, 15]. The major side effects of WT smoking in women are the risk of infection, cancer, lung and respiratory diseases, cardiovascular diseases [5, 16], the risk of low birth weight [17], more premature menopause, decreased bone density, infertility, ectopic pregnancy, increased mortality of infants, and decreased intrauterine growth [16, 18, 19].

Considering the complexity of WT smoking behavior and its relationship with complex social-structural processes, it is better to use special models and theories that include these factors in order to design a suitable intervention for smoking cessation [20, 21]. Since psychosocial issues, beliefs, opinions, physical dependence, subjective norms, low perceived behavioral control are among the determinants of WT smoking in women worldwide [22], it is believed that interventions addressing these factors can facilitate the reduction and cessation of this unhealthy behavior.

The Theory of Planned Behavior (TPB) guided smoking cessation interventions in the past focusing on cigarette smoking [23]. In this present study, we used the Extended Theory of Planned Behavior (ETPB) to guide the design of an educational intervention focusing on WT smoking among women.

### Literature review and theoretical framework

The TPB suggests that a specific behavior (i.e., WT smoking cessation in this study) is predicted by the intention to perform the behavior and perceived behavioral control [24]. Intention of cessation among smokers is a strong predictor of the actual cessation behavior [25]. Perceived behavioral control over smoking cessation is a central mechanism underlying the relationship between nicotine dependence and intention of cessation [26]. Social norms such is another effective factor in smoking cessation [27]. In other words, this theory helps explain how attitude toward the behavior, subjective norm, and perceived behavioral control affect behavior intention and then smoking [28]. When Ajzen developed the TPB, he claimed that the TPB was open to the inclusion of additional predictors [29]. In this study, TPB, was used with an additional construct, habit, as an additional determinant of the intention and behavior of WT cessation.

Habit plays an important role in smoking. Smoking is likely to become a habit during adolescence, and with age, there are higher chances of getting addicted to nicotine [30]. The existing literature showed that habit can be truly effective in smoking behaviors. Besides, habit as an independent variable is a strong predictor of intention [31, 32]. In studies of physical-psychological dependence, habit proved to be an important reason for WT [33, 34]. Currently, there is no conceptual model that takes into account all the above-mentioned variables in smoking cessation.

**Fig. 1:**
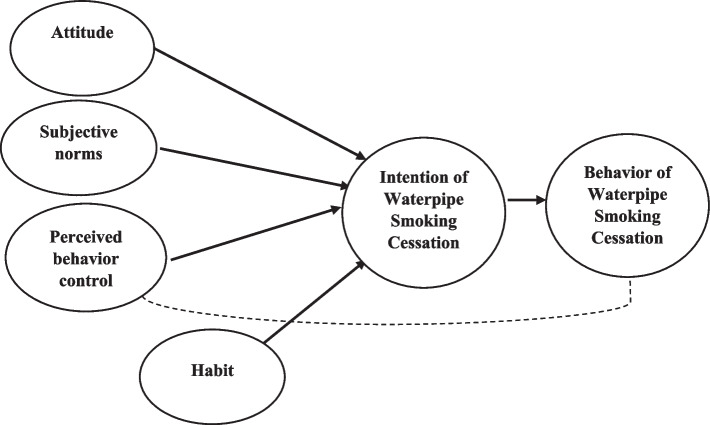
The extended theory of planned behavior

According to the theoretical framework adopted in the present study, behavioral intention consists of willingness to accomplish the behavior. Attitude towards the behavior involves instrumental and experiential appraisals. Perceived behavioral control entails the perceived ability to show the behavior and the confidence to show it. Perceived norms entail the perception of what others do (descriptive norms) and that of other people confirming the behavior (injunctive norms). The main constructs that predict intention are affected by behavioral, normative, and control beliefs that people grow during life [35] (Fig. 1).

Most previous studies have qualitatively and quantitatively identified the factors affecting waterpipe smoking [34, 36, 37]. Limited interventional studies have focused on the behavior of waterpipe smoking, which is the same as the limited studies conducted on the behavior of waterpipe consumption [38] or the intention of cessation [39] and have not attended to the actual behavior of cessation. They also used different models [40, 41] with smaller sample sizes, shorter follow-up and on target populations other than women. To the present researchers’ knowledge, there are not enough theory-based educational interventions on WT cessation behavior of women [31, 42]. Thus, considering the high prevalence of waterpipe smoking in Hormozgan province [43], the present study aimed to determine the effect of an educational intervention based on the ETPB on women’s WT cessation. There are hopes that the present findings help find effective strategies and solutions for WT cessation.

- Hypothesis 1: The scores of ETPB constructs are significantly different between the intervention (IG) and control group (CG) at different times.
- Hypothesis 2: The frequency of waterpipe smoking cessation is different within the IG and CG at different times.

## Methods

### Design and participants

The present quasi-experimental intervention study was conducted with two research groups, a control and an IG on women over 15 years of age who smoked waterpipe in Bandar Abbas city in 2021–2023. Bandar Abbas, the largest port in Iran, located in the north of Hormoz Strait, is the capital of Hormozgan province with a population of 680,366 [44]. This city is bordered by countries in the south of the Persian Gulf.

The inclusion criteria were: 1- the age over 15 years, 2- smoking WT 4 times a week for at least six months as self-reported [45, 46], 3- no history of psychiatric disorders, no history of cardiovascular diseases as self-reported [47], 4- consent to participate in the study, 5- ability to read, write and understand the local or Persian language.

The exclusion criteria were: 1. Simultaneous use of cigarettes or other tobacco products 2. Attempts to cease WT smoking at the same time of the study 3. Simultaneous use of nicotine alternative therapies 4- Participation in other smoking cessation programs 5- Absence of more than two sessions in the training program 6- Not to be available for the post-test.

### Sample size and sampling procedure

As one purpose of the present study was to compare the mean score of perceived behavior control between the IG and CG, to decide on the sample size, the following formula was used to make comparisons between two independent groups:

$$n = \frac{{(z_{{1 - \frac{\alpha }{2}}} + z_{1 - \beta } )^{2} (s_{1}^{2} + s_{2}^{2} )}}{{(\mu_{1} - \mu_{2} )^{2} }}$$

($\mu_{1} - \mu_{2}$) Minimum significant difference between groups = 1

($s_{1}$) Standard deviation in IG = 2.38

($s_{2}$) Standard deviation in CG = 2.55

$$\alpha = 0.05 \to z_{{1 - \frac{\alpha }{2}}} = 1.96$$

$$\beta = 0.2 \to z_{1 - \beta } = 0.84$$

**Fig. 2:**
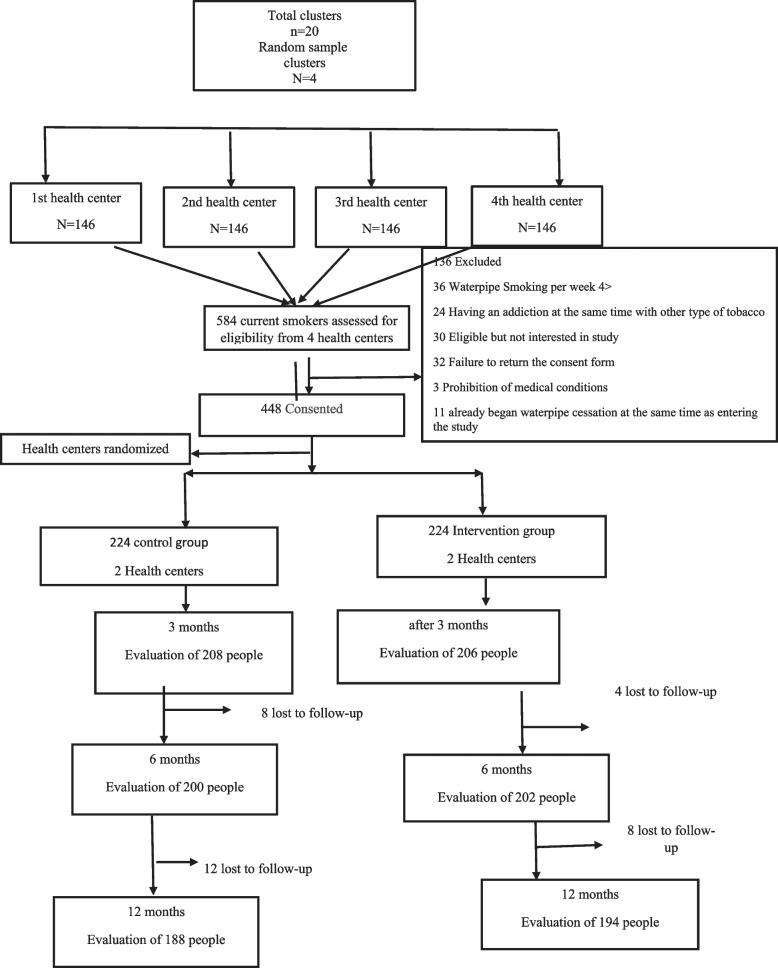
Intervention flowchart

In a similar study by Sotoudeh et al. [48], the standard deviation of mentioned construct in the IG and the CG was, respectively, 2.38 and 2.55. The minimum significant difference in the mean score of the two groups was set at 1. Using the above-mentioned formula, the sample size in each group was estimated at 95. Since the sampling method was clustering, with a design effect of 2, the sample size was estimated at 191 for each group. Considering the duration of the follow-up and an attrition rate of 15%, the final sample size for each group was estimated at 224 with (Fig. 2).

$$n^{\prime} = \frac{n}{1 - \% lost} = \frac{190}{{1 - 0.15}} = 224$$

The sampling method in this study was a two-stage clustering. There are 20 comprehensive health service centers in Bandar Abbas. Four centers with similar socio-economic characteristics were randomly selected among these centers, and two were assigned to the IG and two to the CG. In the next step, the first house from the third alley on the right side of each of the 4 health centers was selected as the head of the cluster, and sampling continued until the cluster was completed. In each household, all eligible members were included in the study. Finally, 448 participants (112 in each of the 4 centers) were contacted after signing a consent form, and 224 in the CG and 224 people in the IG voluntarily completed the questionnaire for the first time (Fig. 2).

### Questionnaire content and scoring system

This questionnaire was researcher-made and had already been used in another study [49].

The questionnaire contained closed-ended questions rated on a Likert scale. There were two parts as introduced below.

#### Part I (Demographic and smoking behavioral information)

This information included age, marital status, educational status, employment status, economic status, the type of tobacco used (local/fruity/both), daily use, weekly use, age of beginning WT smoking, history of WT smoking.

#### Part II (TPB constructs)

##### Knowledge

This variable implied the knowledge of WT cessation and was investigated with 10 items with 3 choices: Yes (1) No (0) I don’t know (0). A sample item is: “The risk of lung cancer in WT smokers is higher than non-smokers.”

##### Attitude

This variable implied one’s thought or feeling about WT cessation. Attitude was measured with 15 items rated on a five-point Likert scale ranging from strongly disagree to strongly agree. A sample item is: “Smoking WTs is dangerous for my health”.

##### Perceived subjective norm

This variable was measured using 20 items to be rated on a five-point Likert scale ranging from strongly disagree to strongly agree. A sample item is: “What my family and friends think about the elimination of tempting signs of WT smoking matters in WT smoking.”

##### Perceived behavioral control

This variable was measured with 10 items rated on five-point Likert scale. A sample item is: “How confident are you that you can break up with WT smoking friends?”.

##### Habit

This variable was measured with 7 items rated on five-point Likert scale ranging from strongly disagree to strongly agree. A sample item is: “Getting used to the pleasure of WT smoking has made it hard for me to quit.”

##### Behavioral intention

This variable was measured with 4 items rated on five-point Likert scale ranging from strongly disagree to strongly agree. A sample item is: “Not in a million years am I going to smoke WTs within the next month.”

##### Behavior

This variable was measured with one item: “Have you ceased smoking WT?” The available choices were YES and NO. If the answer was NO, the respondent was to mention the daily, weekly and monthly frequency of smoking.

### Scoring and reliability

To test the content validity of the measurement instrument, it was provided to 5 experts in health education, 5 doctors trained in smoking cessation and 3 clinical psychologists. Their opinions were used to revise the questionnaire. To test the reliability of the measurement instrument, the test–retest method was used. To this aim, the questionnaire was submitted twice to 30 subjects with similar characteristics to the main participants at two weeks’ interval. Then, each question in the first test was compared with the second test. If the correlation coefficient between the first and second tests in each part of the questionnaire was higher than 0.7, the questionnaire was reliable. In addition, to calculate the agreement between test and retest, the Intraclass correlation coefficient (ICC) was calculated, thus, to calculate the agreement between the mean test scores and the mean retest scores, the ICC value was estimated at 0.833 and the reliability of the questionnaire was substantiated [49].

### Data collection

Data were collected using a self-administered questionnaire at baseline before the intervention, and at 3-, 6- and 12-months follow-up among the two arms of the study. For data collection, the first author visited the first house to begin the sampling in the morning and afternoon. Then the research procedure was explained and a written informed consent form was signed by all the participants. Every participant who consented to take part in the study until the end was given the questionnaire. Each questionnaire took an average of 10 min to complete. Participants completed the questionnaire at home and then returned it to the researcher at the same place. During this process, the researcher checked the questionnaires to remove any potential deficiency. If some questions were not answered, he gave the questionnaire back to the participant for completion). All data were collected before the intervention and in the follow-ups in the IG and CG by the first author during the study to avoid any inter-interviewer disagreement.

### Intervention procedure for the IG

In the present study, the content needed for the educational intervention was obtained from the results of the pre-test (the use of the questionnaire based on ETPB constructs) as well as reliable and academic sources. The educational content of each session as well as educational methods were determined based on the level of learners’ understanding, use of reliable scientific sources, opinions of expert professors, opinions of the participants, and the constituent elements of ETPB. The educational methods in the training sessions were lectures, collaborative discussions, Q&As, brainstorming, role plays, and peer education. A total number of 14 training sessions were held in 6 training groups, each session lasting 90–45 min. The sessions continued from December 5, 2021 to March 13, 2022. The details of the training sessions are provided in Supplementary file 1.

Questionnaires were completed in the CG at the same time as the IG. After the study ended, to comply with the principle of ethics in research and to appreciate the participation of all participants in CG, they also received the materials presented in the educational intervention. CG did not receive any training until the intervention was over.

### Expected outcomes

The primary outcome was WT cessation behavior, which was measured with the question: “Have you ceased WT?” There were two choices available: Yes and No.

By cessation, we mean quitting waterpipe in a 12-month follow-up without any retreat to the WT smoking.

The secondary outcome was the frequency of WT smoking, which was measured by asking the number of times of WT smoking per week for women who did not cease WT smoking (once a day = 1, twice a day = 2, three times a day = 3, more than three times a day = 4).

### Data management and analysis

Mean and standard deviation were used to describe interval variables, and frequency and relative frequency were used to describe non-interval variables. T-test and Chi Square test were used to compare the IG and CG at baseline in terms of demographic variables. Repeated measures ANOVA tests and post hoc tests were used to compare the mean scores of model constructs during the four points of time in the study. Bonferroni correction was used to correct type I error in multiple comparisons. Cochran’s Q test was used to compare the frequency of WT smoking at different times in each of the IG and CG. The data were statistically analyzed in Stata14, and *p*-value < 0.05 was statistically significant. The plots were drawn in MedCalc.

### Ethical considerations

This study was conducted in accordance with the Helsinki guidelines, and a written consent was obtained from all participants. For the participants below 18 years of age, an informed consent was obtained from parents. The ethical approval of this study was jointly obtained from the Research Ethics Committee of Hormozgan University of Medical Sciences (#IR.HUMS.REC.1401.145) and Tehran University of Medical Sciences. The participants were assured of the confidentiality of the information they provided. No fees were charged for participating in the study. Participants at any point of time could withdraw from the study with a written or verbal request.

## Results

The final analysis was done on 194 women in the IG and 188 in the CG. The mean ± SD of the participants’ age in the IG and CG was 39.95 ± 14.65 and 36.60 ± 13.412, respectively. In both groups, most women were married (86.6% of IG, 86.2% of CG). They mostly held a diploma (46.9% of the IG, 58.5% of the control) and had no job (87.1% of the IG, 88.8% of the control). The history of WT smoking in most women (52.6% of the IG, 34.6% of the control) was more than 15 years. The two groups were similar in terms of demographic features and WT smoking behaviors. The only between-group differences were in age and history of WT smoking. Other demographic and behavioral variables of WT smoking and the homogeneity test results of the two groups are presented in Table 1.

**Table 1:** Comparison of demographic and behavioral variables of waterpipe smoking in intervention and control groups

	Intervention group (*n* = 194)	Control group (*n* = 188)	*P*-value
Age (x̄ ± SD)	39.95 ± 14.653	36.60 ± 13.412	.020
Marital status			
Single	26 (13.4%)	26 (13.8%)	.673
Ever married	168 (86.6%)	162 (86.2%)	
Educational level			
< Diploma	82 (42.3%)	64 (34.0%)	.070
Diploma	91 (46.9%)	110 (58.5%)	
University	21 (10.8%)	14 (7.4%)	
Professional activity			
Not working	169 (87.1%)	167 (88.8%)	.606
Working outside home	25 (12.9%)	21 (11.2%)	
Socio-economic status (SES)			
High	68 (35.1%)	61 (32.4%)	.834
Middle	99 (51.0%)	98 (52.1%)	
Low	27 (13.9%)	29 (15.4%)	
Tobacco type			
Local	141 (72.7%)	120 (63.8%)	.106
Fruity	33 (17.0%)	36 (19.1%)	
Both	20 (10.3%)	32 (17.0%)	
Daily smoking			
1	37 (19.1%)	37 (19.7%)	.135
2	38 (19.6%)	25 (13.3%)	
¾	71 (36.6%)	88 (46.8%)	
> 4	48 (24.7%)	38 (20.2%)	
Frequency of waterpipe smoking per week			
0	124 (63.9%)	124 (66.0%)	.846
1–2	45 (23.2%)	39 (20.7%)	
> 2	25 (12.9%)	25 (13.3%)	
WS initiation age			
< 15	43 (22.2%)	37 (19.7%)	.729
15–30	131 (67.5%)	134 (71.3%)	
> 30	20 (10.3%)	17 (9.0%)	
Years of waterpipe smoking			
< 5	51 (26.3%)	60 (31.9%)	.001
5–15	41 (21.1%)	63 (33.5%)	
> 15	102 (52.6%)	65 (34.6%)	

The scores obtained in the intervention and control groups in four points of time, before the intervention, 3, 6 and 12 months after the intervention are shown in Table 2. In the IG, the scores of knowledge, attitude, subjective norms, intention and perceived behavioral control increased while the scores of habit and weekly consumption decreased.

**Table 2:** Comparative mean scores of determinants before intervention, 3, 6 and 12 months after intervention in the control group and intervention group

**Variables**	**Groups**	**Before intervention (x̄ ± SD)**	**3 months after intervention (x̄ ± SD)**	**6 months after intervention (x̄ ± SD)**	**12 months after intervention (x̄ ± SD)**
**Knowledge**	**Intervention**	4.87 ± 2.53	9.09 ± .94	9.37 ± .818	8.68 ± 0.82
	**Control**	4.90 ± 2.57	7.39 ± 1.53	7.77 ± 1.44	7.16 ± 1.40
**Attitude**	**Intervention**	45.79 ± 10.15	59.79 ± 7.33	62.43 ± 10.98	62.62 ± 10.46
	**Control**	45.11 ± 9.83	49.23 ± 9.49	47.48 ± 11.16	47.04 ± 11.36
**Subjective norms**	**Intervention**	61.51 ± 17.26	71.40 ± 15.58	75.15 ± 17.38	74.99 ± 17.14
	**Control**	60.54 ± 18.23	60.97 ± 16.87	57.86 ± 19.26	57.66 ± 19.16
**Habit**	**Intervention**	25.61 ± 6.76	17.13 ± 7.63	15. 50 ± 8. 98	14.82 ± 7.83
	**Control**	24.81 ± 5.86	28.85 ± 5.35	32. 39 ± 5. 43	32.74 ± 4.73
**Intention**	**Intervention**	10. 69 ± 4. 78	16. 01 ± 2. 23	16.32 ± 3. 36	16. 66 ± 2.94
	**Control**	11. 21 ± 4. 46	10. 99 ± 4. 60	12.25 ± 4. 75	11. 56 ± 4.48
**Perceived behavioral control**	**Intervention**	40.84 ± 17.84	62.57 ± 11.23	64.45 ± 12.88	65.00 ± 12.59
	**Control**	40.81 ± 18.36	40.89 ± 14.88	40.93 ± 12.06	41.33 ± 11.36
**Frequency of waterpipe smoking per week**	**Intervention**	22.41 ± 15.55	3.98 ± 6.70	3.39 ± 5.69	3.093 ± 5.40
	**Control**	23.60 ± 19.59	19.67 ± 17.02	20.64 ± 16.09	22.00 ± 16.33

Comparison of the two groups in 4 points of time (i.e., before intervention, 3, 6 and 12 months after intervention) shows that the mean score of the determinants of WT smoking in the two groups at time 1 (before the intervention) was not significantly different (*p*-value > 0.999, intention *p*-value = 0.830). However, there was a statistically significant difference between the two groups 3, 6 and 12 months after the intervention (*p*-value < 0.001) (Table 3).

**Table 3:** Comparative mean differences of determinants before intervention, 3, 6 and 12 months after intervention vs. control

	**Time**	**Mean difference**	**95% confidence interval**	***P*-value**
Knowledge	**(Intervention VS Control)1**	-0.33	**-0.45 to 0.39**	> 0.999
	**(Intervention VS Control)2**	1.69	**1.27 to 2.11**	< 0.001
	**(Intervention VS Control)3**	1.56	**1.18 to 2.02**	< 0.001
	**(Intervention VS Control)4**	1.52	**1.10 to 1.94**	< 0.001
Attitude	**(Intervention VS Control)1**	0.68	**-1.92 to 3.28**	> 0.999
	**(Intervention VS Control)2**	10.56	**7.96 to 13.17**	< 0.001
	**(Intervention VS Control)3**	14.95	**12.35 to 17.55**	< 0.001
	**(Intervention VS Control)4**	15.58	**12.97 to 18.18**	< 0.001
Subjective norms	**(Intervention VS Control)1**	0.97	**-3.55 to 5.48**	> 0.999
	**(Intervention VS Control)2**	10.43	**5.92 to 14.95**	< 0.001
	**(Intervention VS Control)3**	17.29	**12.77 to 21.80**	< 0.001
	**(Intervention VS Control)4**	17.33	**12.82 to 21.84**	< 0.001
Habit	**(Intervention VS Control)1**	0.79	**-0.93 to 2.52**	0.996
	**(Intervention VS Control)2**	-11.72	**-13.44 to—9.10**	< 0.001
	**(Intervention VS Control)3**	-16.89	**-18.61 to -15.16**	< 0.001
	**(Intervention VS Control)4**	-17.92	**-19.64 to -16.20**	< 0.001
Intention	**(Intervention VS Control)1**	-0.52	**-1.56 to 0.51**	0.830
	**(Intervention VS Control)2**	5.02	**3.98 to 6.06**	< 0.001
	**(Intervention VS Control)3**	4.06	**3.03 to 5.10**	< 0.001
	**(Intervention VS Control)4**	5.09	**4.06 to 6.13**	< 0.001
Perceived behavioral control	**(Intervention VS Control)1**	0.03	**-3.59 to 3.65**	> 0.999
	**(Intervention VS Control)2**	21.68	**18.06 to 25.30**	< 0.001
	**(Intervention VS Control)3**	23.52	**19.90 to 27.14**	< 0.001
	**(Intervention VS Control)4**	23.67	**20.05 to 27.29**	< 0.001
Frequency of waterpipe smoking per week	**(Intervention VS Control)1**	-1.19	**-4.74 to 2.35**	> 0.999
	**(Intervention VS Control)2**	-15.70	**-19.24 to -12.15**	< 0.001
	**(Intervention VS Control)3**	-17.25	**-20.79 to -13.71**	< 0.001
	**(Intervention VS Control)4**	-18.91	**-22.45 to -15.36**	< 0.001

The within-group comparison of different constructs in 4 points of time (i.e., before intervention, 3, 6 and 12 months after intervention) shows that after 12 months of educational intervention in the IG, there was an increase (*p*-value < 0.001), respectively, in perceived behavioral control, attitude, subjective norms, intention and knowledge (*p*-value < 0.001). There was a decrease in weekly WT smoking and habit (*p*-value < 0.001). The control group also had no significant change in other variables except an increase in the knowledge score. Other details including the between-group comparison of scores across the four points of time are presented in Supplementary file 2.

**Fig. 3:**
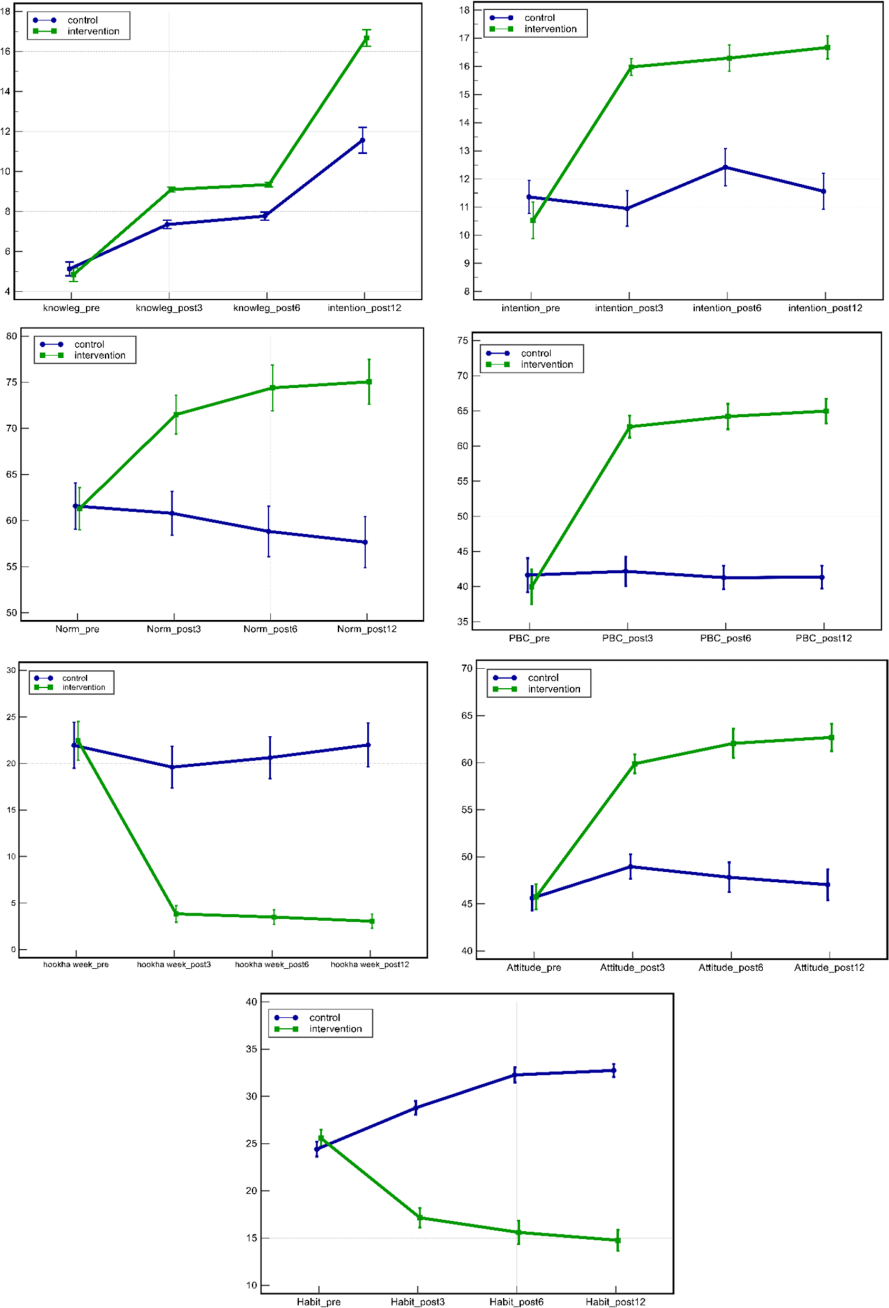
Comparison of determinants of cessation before intervention, 3, 6 and 12 months after intervention in the two research groups

Figure 3 shows the comparison of the determinants of WT cessation before the intervention, 3, 6 and 12 months after the intervention in the two groups. The IG had an increase in PBC, attitude, subjective norms, intention and knowledge compared to the time before the intervention. A similar increase was also found in the IG compared to the control group. A decrease was observed in habit and weekly smoking of the IG.

The results of the Cochran’s test showed that in the IG, after 3 months, 48.45% of the participants managed to cease smoking WTs. In the subsequent follow-ups, the return to smoking was not statistically significant (*p*-value = 0.645). This showed the effectiveness of the educational intervention in the successful behavior of WT smoking cessation (Table 4).

**Table 4:** Distribution of waterpipe smoking 3, 6 and 12 months after intervention in the two research groups

Group	Point of time	Ceased	Not ceased	Cochran’s Q	*P*-value
Intervention	Waterpipe-smoking_post3	94 (48.45)	100 (51.55)	0.787	0.645
	Waterpipe-smoking _post6	90 (46.39)	104 (53.61)		
	Waterpipe-smoking _post12	85 (43.81)	109 (56.19)		
Control	Waterpipe-smoking _post3	27 (14.36)	161 (85.64)	5.019	0.081
	Waterpipe-smoking _post6	18 (9.57)	170 (90.43)		
	Waterpipe-smoking _post12	14 (7.45)	174 (92.55)		

## Discussion

The present research aimed to explore the effect of a TPB-based educational intervention on WT smoking cessation in women. The educational intervention managed to improve the perceived behavioral control, attitude, subjective norms, intention and knowledge in the IG and reduce the habit and weekly smoking of WT.

The present findings showed that the mean knowledge score after the intervention in the IG was higher than the control. There was a statistically significant difference between the two groups in all points of time after the intervention. Similarly, in another study in Iran, the researchers reported that the mean knowledge score increased after a training campaign which positively affected the WT smoking behavior [50]. In other similar studies, educational interventions managed to improve knowledge [51, 52]. Of note is that in both the intervention and control groups, the mean knowledge score increased after the intervention. However, the increased knowledge of the control group could not affect women’s WT smoking behavior. It can be argued that in the IG, the increased knowledge of the benefits of cessation and the impact of WT on health, along with other determinants of WT cessation managed to positively affect the reduction and cessation of WT smoking. Probably the mere increase of knowledge does not suffice, and other effective factors should also be considered, including attitude, subjective norms, perceived behavioral control and habit.

As the present findings showed, the educational intervention managed to change women’s attitude. Thus, a positive attitude towards smoking cessation and a negative attitude towards WT smoking were observed in women of the IG. What people think and believe plays an important role in their act of smoking, and the more the consequences of smoking are perceived as positive, the higher the chances of people smoking [53]. Still, we can claim we can change people’s attitudes when this change leads to a change in their behavior. In the present research, ceasing the behavior and the rate of WT smoking were investigated, and considering the rate of cessation and reduction of WT smoking, we can say that the educational intervention has been partly successful in changing women’s attitudes towards WT smoking. According to Ajzen, a change in attitude leads to a change in intention, which in turn affects behavior [54]. In line with this finding, Alalwan also stated that an increasing negative attitude towards WT smoking is associated with a high motivation to cease smoking [55].

The present findings showed that the mean score of WT habit in the IG decreased after the educational intervention compared to the control. Also, the findings concerning habit showed that as we moved away from the intervention time, the participants’ dependence on WT smoking in the IG decreased. This is indicative of the fact that just as a habit or a physical-psychological dependence on WT is formed over time, it takes time to eliminate it [56, 57]. The classification of smoking cues includes internal, habitual, social, and environmental cues. The habitual cues represent prior classical conditioning. To break the relationship between habitual activity and smoking behavior, the expectancy of smoking can be reduced. To develop or assess smoking cessation programs, the Theory of Planned Behavior (TPB) has been used, with a particular focus on behavioral intentions, attitudes, subjective norms, and perceived behavioral control related to smoking cessation among women. The TPB proved to be truly effective predictor for the behavior of smoking [58]. The present researchers contend that breaking the habit can be an important factor in the success of WT smoking cessation. Therefore, it seems that the recognition of all variables that contribute to habit-formation and making some intervention in them help reduce or cease the habit of WT smoking.

The present research showed that the mean score of PBC in relation to WT cessation increased in the IG after the educational intervention compared to the control. This between-group difference was statistically significant. Consistent with this finding, the results of Joveini’s study showed that an educational intervention in light of TPB led to more trust and confidence in participants to control conditions to cease WT smoking and deal with the tempting conditions of WT smoking [39]. Arguably, people who have clear, well-defined, coordinated and almost stable perceived behavioral control have higher psychological health; therefore, it is less likely that they take an unhealthy measure to solve their problem, such as WT smoking. In this regard, Hagger reported in a meta-analysis that PBC moderated the intention-behavior relationship in health-related behaviors [59]. Contrary to these findings, Bashirian et al. contended that the difference in PBC score was not statistically significant between the intervention and control groups [60]. The possible cause of this difference can be attributed to both the type of intervention and the individuals participating in the study. The researchers in the afore-mentioned study used a web-based intervention and the participants were adolescent girls.

The present findings showed a significant increase in the mean score of subjective norms in the IG compared to the control. It seems that significant others’ advice is a critical factor in reducing and ceasing WT smoking. Besides, the motivation to follow, an indirect construct of subjective norms, can play a major role in changing women’s behavior of WT smoking. In traditional societies, where the role of culture and influential others is effective in forming individual behavior, interventions based on subjective norms are more effective, and in the existing literature, such interventions have been effective on tobacco behavior [61, 62]. In this regard, Najafi in Iran reported an improvement in the mean score of subjective norms in WT women smokers in the IG compared to the control [63]. It might be claimed that an important factor that can encourage one to cease WT smoking is the strong motivation provided by important and influential others in one’s life [64]. In another study, the relationship between motivation to follow and smoking cessation was also proven [65]; therefore, using this strategy means to engage important and influential others in women’s lives and to persuade them to positively and significantly affect WT cessation.

Another finding of the present study was the increased mean score of behavioral intention in the IG after the educational intervention compared to the control. It seems that increasing knowledge of the side effects of WT smoking, the physical benefits of cessation, positive attitude towards cessation, and following influential others’ advice in life lead to an increase in women’s intention to cease WT smoking. In line with the present study, other researchers also contended that after educational interventions, there was an increase in the intention to cease smoking and a decrease in WT smoking in the IG [39, 66]. In the present study, all participants who intended to cease WT smoking did not succeed. It seems that there were barriers to translating intention into behavior, or in other words, other facilitators were needed to turn intention into behavior. In fact, these factors act as intermediary factors that make it possible to translate intention into behavior, and their absence can disrupt this process [67]. It is necessary to investigate and overcome these barriers in future studies to increase the rate of WT cessation as far as possible.

The findings also showed the performance of WT cessation behavior and reduced weekly smoking in the IG after the intervention compared to before and also in comparison to the control group. This finding confirms that the TPB has been effective in changing the behavior of WT smoking. It seems that we managed to influence the determinants of WT smoking, which takes into account the aspects of personal and interpersonal behavior, to change women’s WT smoking behavior in the IG. In line with the present findings, other studies also reported a reduction in WT smoking after the educational intervention, in the IG compared to the control [40, 66]. Other studies such as Rajabalipour’s research conducted in light of the socio-cognitive theory also showed although the scores of some constructs changed significantly after the intervention, no significant change was observed in the prevalence of WT smoking [41]. This contradiction can be partially attributed to the type of educational intervention design, considering the different models used, the duration of intervention, and the demographic features of the participants. To explain why the rate of WT smoking decreased but not completely ceased, it seems that women were not able to overcome the physical and psychological dependence (habit) on WT smoking. Also, it may be possible to acknowledge that in addition to personal factors, external factors also influence the WT smoking behavior, which are out of the participants’ and researchers’ control [34]. Researchers need to pay more attention to factors affecting WT smoking in future research.

### Limitations

Sampling women in one city of Hormozgan province probably limits the generalizability of findings to women in other provinces and other target populations, including men. However, to increase generalizability, the sampling was done in the largest city of the province and among women with diverse socio-demographic features. Therefore, the findings can be cautiously generalized to women in southern Iran and regions of the Middle East with similar cultural backgrounds. Failure to perform biochemical tests to confirm smoking cessation was another limitation of the present study, although from the beginning, attempts were made to reduce the effect of this bias by increasing trust and intimacy between the researcher and participants. In this regard, studies of truthfulness in describing the real condition of WT smoking after the educational intervention confirmed the establishment of more trust and intimacy than before the intervention [41, 68]. The appropriate sample size and 12-month follow-up to evaluate the rate of return to WT-smoking behavior was one of the strengths of this research.

### Implications

Considering the high costs imposed by the physical and psychological effects of WT smoking, the present findings can provide useful information to health policymakers to set standards and guidelines for to bacco smoking cessation, including WT smoking. It is recommended to use this program not only throughout Iran but also worldwide in other regions that need a tried and tested WT cessation program.

## Conclusion

As the present findings showed, women’s belief and will to cease WT smoking successfully as well as influential others’ advice in life played a significant role in reducing and ceasing WT smoking in women. The educational intervention had a positive effect on the reduction and cessation of WT smoking in women through influencing the TPB constructs. It is strongly recommended to develop theory-based interventions beyond the personal level with an emphasis on interpersonal relationships in order to facilitate WT cessation as far as possible.

### Supplementary Information

**Supplementary Material 1.**

**Supplementary Material 2.**

**Supplementary Material 3.**

## Data Availability

The datasets generated during and/or analyzed during the current study are available from the corresponding author on reasonable request.

## Abbreviations

PBC: Perceived behavior control
TPB: Theory of Planned Behavior
WT: Waterpipe
CG: Control group
IG: Intervention group
ETPB: Extended Theory of Planned Behavior
