# Supplementary material for: The effectiveness of a theory -based health education program on waterpipe smoking cessation in Iran: one year follow-up of a quasi-experimental research
Source: BMC Public Health. 2024 Mar 1;24:664. doi: 10.1186/s12889-024-18169-7 (PMC10908080; doi:10.1186/s12889-024-18169-7)
Supplement: Supplementary file 1 — Supplementary Material 1. [file 12889_2024_18169_MOESM1_ESM.docx]

**Supplementary file 1. Education and training content**

| **Session no** | **Time** | **Topic** | **Mentioned Items** |
| --- | --- | --- | --- |
| 1 | 60 min | Knowledge / Perceived behavior control: HTS adverse effects and temptations | In this session, women were familiarized with the adverse effects and tempting factors of HTS with help of the instructor through lecture, brain-storming, discussion and active participation. Also, women were familiarized with how to stay away from the tempting HTS conditions with help of the instructor through lecture, active participatory discussions using the role model. |
| 2 | 60 min | Perceived behavior control: Significance and essentiality of HTS cessation | In this session, the role model was used to invite people who got in trouble due to HTS. Besides, a movie was shown to display HTS adverse effects and compare women smokers and non-smokers to further highlight the significance and essentiality of HTS cessation in women. |
| 3 | 90 min | Knowledge / Attitute: Barriers to HTS cessation, short- and long-term adverse effects of HTS | In this session, women got to know about the adverse effects of HTS with help of the instructor through active participatory discussions, brain-storming, movie show (“death Symphony”), observation of women smokers’ and non-smokers’ faces, and a video on physicians’ comments shown in mass media on the adverse effects of HTS and its mortality rate and concomitant diseases. Then, they got to know about the benefits of HTS cessation through an educational pamphlet, evaluating the barriers of HTS cessation, and learned about the short-term and long-term adverse effects of HTS. |
| 4 | 45 min | Social norms: Significance of HTS cessation among women | In this session, the supporters got to know the role model, and the significance and essentiality of HTS cessation and emotional supports along the cessation pathway. |
| 5 | 45 min | Social norms/ Perceived behavior control: Useful cessation strategies among women | In this session, the supporters learned about different useful and effective strategies of limiting HTS inside or outside home with help of the instructor through brain-storming and participatory discussions. |
| 6 | 45 min | Attitude: Significance of emotional and social support of women | In this session, the supporters learned about the significant role of emotional support with the help of the instructor and through lecture, brain-storming and active participatory discussion. |
| 7 | 45 min | Intention: Effective HTS cessation strategies | In this session, women were familiarized with different useful and effective strategies about how to stay away from HTS peers and contaminated areas, with help of the instructor and the role model. |
| 8 | 45 min | Perceived behavior control: How to resist HTS temptation | In this session, the women got to know how to resist hookah temptations with the help of instructor and the role model, through guided practice and participatory discussions. |
| 9 | 45 min | Habit: HS habit cessation | In this session, participatory discussion and brainstorming were used to familiarize women with alternative HTS activities and how to stay away from HTS tempting conditions. |
| 10 | 45 min | Habit: HS habit cessation | In this session, participatory discussion and brainstorming were used to enlist barriers to HS cessation. Then, suggestions were made on how to eliminate these barriers through brainstorming. |
| 11 | 45 min | Intention / Attitude: Risks and outcomes of HS | In this session, participatory discussion and brainstorming were used with the help of a physician to familiarize women with the outcomes of HS. |
| 12 | 45 min | Intention / Perceived behavior control: How to prevent and react appropriately in the case of recurrence | In this session, participatory discussion and brainstorming were used in the presence of a clinical psychologist to familiarize women with how to prevent and react appropriately to the recurrence of the old habit. |
| 13 | 60 min | Perceived behavior control: How to control external factors and resist HS temptation | In this session, participatory discussion and brainstorming were used in the presence of a clinical psychologist to familiarize women with how to control external stimuli. the clinical psychologist familiarized women with how to resist HS temptations. |
| 14 | 90 min | Attitude/ Perceived behavior control: Motivation for cessation | In this session, participatory discussion and brainstorming were used in the presence of a clinical psychologist to familiarize women with how to increase self-efficacy. Also, the clinical psychologist familiarized women with the physical and social benefits of HS cessation and helped further motivate women to cease HS. |
