# Supplementary material for: The effectiveness of a theory -based health education program on waterpipe smoking cessation in Iran: one year follow-up of a quasi-experimental research
Source: BMC Public Health. 2024 Mar 1;24:664. doi: 10.1186/s12889-024-18169-7 (PMC10908080; doi:10.1186/s12889-024-18169-7)
Supplement: Supplementary file 2 — Supplementary Material 2. [file 12889_2024_18169_MOESM2_ESM.docx]

**Table 4: Pairwise comparisons of TBP scores and at different times with each other for two groups**

|  | **groups** | **times** | **Mean difference** | **95% confidence interval** | **p-value** |
| --- | --- | --- | --- | --- | --- |
| **knowledge** | **control** | **2 VS 1** | 2.49 | **2.05 to 2.93** | <0.001 |
|  |  | **3 VS 1** | 2.87 | **2.42 to 3.31** | <0.001 |
|  |  | **4 VS 1** | 2.25 | **1.81 to 2.70** | <0.001 |
|  |  | **3 VS 2** | 0.38 | **-0.07 to 0.82** | 0.148 |
|  |  | **4 VS 2** | -0.23 | **-0.68 to 0.21** | 0.980 |
|  |  | **4 VS 3** | -0.61 | **-1.05 to -0.17** | 0.002 |
|  | **intervention** | **2 VS 1** | 4.22 | **3.78 to 4.65** | <0.001 |
|  |  | **3 VS 1** | 4.5 | **4.06-4.94** | <0.001 |
|  |  | **4 VS 1** | 3.81 | **3.37 to 4.25** | <0.001 |
|  |  | **3 VS 2** | 0.28 | **-0.15 to 0.72** | 0.518 |
|  |  | **4 VS 2** | -0.41 | **-0.84 to 0.03** | 0.083 |
|  |  | **4 VS 3** | -0.69 | **-1.13 to -0.25** | 0.000 |
| **attitude** | **control** | **2 VS 1** | 4.12 | **1.42 to 6.82** | <0.001 |
|  |  | **3 VS 1** | 2.37 | **-0.33 to 5.07** | 0.123 |
|  |  | **4 VS 1** | 1.94 | **-0.76 to 4.64** | 0.351 |
|  |  | **3 VS 2** | -1.75 | **-4.45 to 0.95** | 0.523 |
|  |  | **4 VS 2** | -2.19 | **-4.89 to 0.51** | 0.196 |
|  |  | **4 VS 3** | -0.44 | **-3.14 to 2.26** | >0.999 |
|  | **intervention** | **2 VS 1** | 14.00 | **11.34 to 16.66** | <0.001 |
|  |  | **3 VS 1** | 16.64 | **13.98 to 19.30** | <0.001 |
|  |  | **4 VS 1** | 16.83 | **14.17 to 19.49** | <0.001 |
|  |  | **3 VS 2** | 2.64 | **0.02 to 5.30** | 0.053 |
|  |  | **4 VS 2** | 2.82 | **0.16 to 5.48** | 0.031 |
|  |  | **4 VS 3** | 0.18 | **2.47 to 2.84** | >0.999 |
| **Social norm** | **control** | **2 VS 1** | 0.43 | **-4.31 to 5.17** | >0.999 |
|  |  | **3 VS 1** | -2.68 | **-7.42 to 2.06** | 0.813 |
|  |  | **4 VS 1** | -2.88 | **-7.62 to 1.86** | 0.651 |
|  |  | **3 VS 2** | -3.11 | **-7.85 to 1.63** | 0.499 |
|  |  | **4 VS 2** | -3.31 | **-8.05 to 1.43** | 0.390 |
|  |  | **4 VS 3** | -0.20 | **-4.94 to 4.54** | >0.999 |
|  | **intervention** | **2 VS 1** | 9.90 | **5.23 to 14.56** | <0.001 |
|  |  | **3 VS 1** | 13.64 | **8.97 to 18.31** | <0.001 |
|  |  | **4 VS 1** | 13.48 | **8.81 to 18.15** | <0.001 |
|  |  | **3 VS 2** | 3.74 | **-0.93 to 8.41** | 0.206 |
|  |  | **4 VS 2** | 3.58 | **-1.09 to 8.25** | 0.257 |
|  |  | **4 VS 3** | -0.16 | **-4.83 to 4.51** | >0.999 |
| **Habit** | **control** | **2 VS 1** | 4.04 | **2.20 to 5.88** | <0.001 |
|  |  | **3 VS 1** | 7.58 | **5.74 to 9.42** | <0.001 |
|  |  | **4 VS 1** | 7.93 | **6.09 to 9.77** | <0.001 |
|  |  | **3 VS 2** | 3.54 | **1.70 to 5.38** | <0.001 |
|  |  | **4 VS 2** | 3.89 | **2.05 to 5.73** | <0.001 |
|  |  | **4 VS 3** | 0.35 | **-1.49 to 2.19** | >0.999 |
|  | **intervention** | **2 VS 1** | -8.48 | **10.29 to -6.67** | <0.001 |
|  |  | **3 VS 1** | -10.10 | **-11.92 to -8.29** | <0.001 |
|  |  | **4 VS 1** | -10.78 | **-12.60 to -8.97** | <0.001 |
|  |  | **3 VS 2** | -1.62 | **-3.44 to 0.19** | 0.109 |
|  |  | **4 VS 2** | -2.30 | **-4.12 to -0.49** | 0.005 |
|  |  | **4 VS 3** | -0.68 | **-2.49 to 1.13** | >0.999 |
| **Intention** | **control** | **2 VS 1** | -0.22 | **-1.32 to 0.88** | >0.999 |
|  |  | **3 VS 1** | 1.042 | **-0.06 to 2.14** | 0.074 |
|  |  | **4 VS 1** | 0.351 | **-0.75 to 1.45** | >0.999 |
|  |  | **3 VS 2** | 1.261 | **0.16 to 2.36** | 0.015 |
|  |  | **4 VS 2** | 0.5691 | **-0.53 to 1.67** | >0.999 |
|  |  | **4 VS 3** | -0.691 | **-1.79 to 0.41** | 0.579 |
|  | **intervention** | **2 VS 1** | 5.325 | **4.24 to 6.41** | <0.001 |
|  |  | **3 VS 1** | 5.629 | **4.55 to 6.71** | <0.001 |
|  |  | **4 VS 1** | 5.969 | **4.89 to 7.05** | <0.001 |
|  |  | **3 VS 2** | 0.304 | **-0.78 to 1.39** | >0.999 |
|  |  | **4 VS 2** | 0.644 | **-0.44 to 1.73** | 0.694 |
|  |  | **4 VS 3** | 0.340 | **-0.74 to 1.42** | >0.999 |
| **Perceived behavioral control** | **control** | **2 VS 1** | 0.08 | **-3.52 to 3.68** | >0.999 |
|  |  | **3 VS 1** | 0.12 | **-3.48 to 3.72** | >0.999 |
|  |  | **4 VS 1** | 0.52 | **-3.08 to 4.12** | >0.999 |
|  |  | **3 VS 2** | 0.04 | **-3.56 to 3.64** | >0.999 |
|  |  | **4 VS 2** | 0.44 | **-3.16 to 4.04** | >0.999 |
|  |  | **4 VS 3** | 0.40 | **-3.20 to 4.00** | >0.999 |
|  | **intervention** | **2 VS 1** | 21.73 | **18.19 to 25.28** | <0.001 |
|  |  | **3 VS 1** | 23.61 | **20.07 to 27.16** | <0.001 |
|  |  | **4 VS 1** | 24.16 | **20.61 to 27.70** | <0.001 |
|  |  | **3 VS 2** | 1.88 | **-1.66 to 5.43** | 0.966 |
|  |  | **4 VS 2** | 2.43 | **-1.12 to 5.97** | 0.423 |
|  |  | **4 VS 3** | 0.55 | **-2.99 to 4.091** | >0.999 |
| **Frequency of HS per week** | **control** | **2 VS 1** | -3.92 | **-7.60 to -0.24** | 0.029 |
|  |  | **3 VS 1** | -2.96 | **-6.64 to 0.72** | 0.201 |
|  |  | **4 VS 1** | -1.60 | **-5.28 to 2.08** | >0.999 |
|  |  | **3 VS 2** | 0.96 | **-2.72 to 4.64** | >0.999 |
|  |  | **4 VS 2** | 2.32 | **-1.35 to 6.00** | 0.572 |
|  |  | **4 VS 3** | 1.36 | **-2.32 to 5.042** | >0.999 |
|  | **intervention** | **2 VS 1** | -18.43 | **-22.05 to -14.80** | <0.001 |
|  |  | **3 VS 1** | -19.02 | **-22.64 to -15.40** | <0.001 |
|  |  | **4 VS 1** | -19.31 | **-22.94 to -15.69** | <0.001 |
|  |  | **3 VS 2** | -0.59 | **-4.21 to 3.03** | >0.999 |
|  |  | **4 VS 2** | -0.89 | **-4.51 to 2.74** | >0.999 |
|  |  | **4 VS 3** | -0.29 | **-3.92 to 3.33** | >0.999 |
