# Supplementary material for: The effectiveness of a theory -based health education program on waterpipe smoking cessation in Iran: one year follow-up of a quasi-experimental research
Source: BMC Public Health. 2024 Mar 1;24:664. doi: 10.1186/s12889-024-18169-7 (PMC10908080; doi:10.1186/s12889-024-18169-7)
Supplement: Supplementary file 3 — Supplementary Material 3. [file 12889_2024_18169_MOESM3_ESM.docx]

**Behavior**

Have you ceased smoking WT?

YES NO

If the answer is NO

Current state of waterpipe smoking:

1. How often do you smoke waterpipe on a daily basis? ........... times a day
2. How often do you smoke waterpipe on a weekly basis? ........... times a week
3. How often do you smoke waterpipe on a monthly basis? ........... times a month

**Knowledge**

1. Those who smoke waterpipe are more likely to get mouth, tongue and gum cancer.

True Ο False Ο Don’t Know Ο

1. The risk of lung cancer is higher in waterpipe smokers than in non-smokers.

True Ο False Ο Don’t Know Ο

1. Waterpipe is less harmful than cigarettes and other tobacco products.

True Ο False Ο Don’t Know Ο

1. Waterpipe smoke is purified by the water tank and is not harmful.

True Ο False Ο Don’t Know Ο

1. Waterpipe is not addictive like cigarettes, so it is very easy to quit.

True Ο False Ο Don’t Know Ο

1. Amusing yourself with various activities (distractions) can be effective in quitting it successfully.

True Ο False Ο Don’t Know Ο

1. After quitting waterpipe, new conditions appear (depressed mood, insomnia, anxiety, increased appetite, restlessness), which are quite common.

True Ο False Ο Don’t Know Ο

1. Adhering to smoking waterpipe reduces mental-psychological complications in people.

True Ο False Ο Don’t Know Ο

9. Staying away from environments contaminated with waterpipe smoking can be effective in quitting it successfully.

True Ο False Ο Don’t Know Ο

10. Announcing the decision to quit waterpipe to those around me can be effective in quitting waterpipe.

True Ο False Ο Don’t Know Ο

|  | **Attitude** | | | | | |
| --- | --- | --- | --- | --- | --- | --- |
| no |  | Strongly agree | Agree | Undecided | Disagree | Strongly disagree |
| 1 | Ceasing waterpipe smoking is beneficial for me. |  |  |  |  |  |
| 2 | The advantages of smoking waterpipe are more than disadvantages. |  |  |  |  |  |
| 3 | Ceasing waterpipe smoking requires a lot of effort and energy. |  |  |  |  |  |
| 4 | Smoking waterpipe is dangerous for my health. |  |  |  |  |  |
| 5 | It is important for me to identify the tempting conditions of waterpipe smoking. |  |  |  |  |  |
| 6 | I feel good about staying away from the tempting conditions of waterpipe smoking. |  |  |  |  |  |
| 7 | I prefer healthy entertainment to smoking waterpipes. |  |  |  |  |  |
| 8 | I’d better quit waterpipe smoking as soon as possible. |  |  |  |  |  |
| 9 | I don't believe in the side effects of waterpipe smoking. That's why it doesn't matter when I quit. |  |  |  |  |  |
| 10 | I compare and pay attention to the positive and negative changes of smoking waterpipes. |  |  |  |  |  |
| 11 | The positive changes of smoking waterpipes are more important than the negative changes. |  |  |  |  |  |
| 12 | I don't care about the side effects of smoking waterpipes. |  |  |  |  |  |
| 13 | If I made a mistake in favor of smoking waterpipes during the cessation, this means failure in cessation. |  |  |  |  |  |
| 14 | Not interacting with waterpipe smokers has many benefits for me. |  |  |  |  |  |
| 15 | I feel good about breaking up with waterpipe smoking friends. |  |  |  |  |  |
|  | **Subjective norms** | Totally important to me | Important to me | Undecided | Unimportant to me | Totally unimportant to me |
| 1 | What my family and relatives think about waterpipe cessation |  |  |  |  |  |
| 2 | What my friends think about waterpipe cessation |  |  |  |  |  |
| 3 | What my family and relatives think about replacing waterpipe smoking with healthy entertainments |  |  |  |  |  |
| 4 | What my friends think about replacing waterpipe smoking with healthy entertainments |  |  |  |  |  |
| 5 | What my family and relatives think about not socializing with waterpipe smokers |  |  |  |  |  |
| 6 | What my friends think about not socializing with waterpipe smokers |  |  |  |  |  |
| 7 | What my family and relatives think about not visiting tempting places |  |  |  |  |  |
| 8 | What my friends think about not visiting tempting places |  |  |  |  |  |
| 9 | What my family and relatives think about identification of stimulants of waterpipe smoking |  |  |  |  |  |
| 10 | What my friends think about identification of stimulants of waterpipe smoking |  |  |  |  |  |
| 11 | What my family and relatives think about omitting any tempting factor of waterpipe smoking |  |  |  |  |  |
| 12 | What my friends think about omitting any tempting factor of waterpipe smoking |  |  |  |  |  |
| 13 | My family and relatives expecting me to quit waterpipe smoking |  |  |  |  |  |
| 14 | My friends expecting me to quit waterpipe smoking |  |  |  |  |  |
| 15 | My family and relatives expecting me to replace smoking with other healthy behaviors |  |  |  |  |  |
| 16 | My friends expecting me to replace smoking with other healthy behaviors |  |  |  |  |  |
| 17 | My family and relatives expecting me to stop seeing waterpipe smoking friends |  |  |  |  |  |
| 18 | My friends expecting me to stop seeing waterpipe smoking friends |  |  |  |  |  |
| 19 | My family and relatives expecting me to stop visiting contaminated and tempting places (to smoking) |  |  |  |  |  |
| 20 | My friends expecting me to stop visiting contaminated and tempting places (to smoking) |  |  |  |  |  |
|  | **Habit** | | | | | |
|  | Item | Strongly agree | Agree | Undecided | disagree | Strongly disagree |
| 1 | I can't quit waterpipe because of smoking for many years. |  |  |  |  |  |
| 2 | I smoke waterpipe because it's a long-held habit and is hard to quit. |  |  |  |  |  |
| 3 | As I am used to smoking waterpipes, I make excuses to quit it. |  |  |  |  |  |
| 4 | Getting used to the pleasure of smoking waterpipes has made it hard for me to quit. |  |  |  |  |  |
| 5 | Psychological dependence on waterpipe makes it hard to quit. |  |  |  |  |  |
| 6 | Physical dependence on waterpipe makes it hard to quit. |  |  |  |  |  |
| 7 | I smoke waterpipe because I am addicted to it. |  |  |  |  |  |
| **Intention** | | | | | | |
| 1 | I absolutely do not aim to smoke waterpipes even once in the next week. |  |  |  |  |  |
| 2 | I absolutely do not aim to smoke waterpipes even once in the next month. |  |  |  |  |  |
| 3 | I absolutely do not aim to smoke waterpipes even once in the next three months. |  |  |  |  |  |
| 4 | I absolutely do not aim to smoke waterpipes even once in the next six months. |  |  |  |  |  |

**Perceived behavioral control questions**:

Please choose the best fitting answer from 1 to 10 based on your current condition. 1 shows the least confidence, and as you move to larger values, it shows more confidence. Thus, 10 represents the highest confidence.

1. How confident are you that you can quit waterpipe despite external stimuli?

| **10** | **9** | **8** | **7** | **6** | **5** | **4** | **3** | **2** | **1** |
| --- | --- | --- | --- | --- | --- | --- | --- | --- | --- |

**2.** How confident are you that you can deal with the temptations of waterpipe use?

| **10** | **9** | **8** | **7** | **6** | **5** | **4** | **3** | **2** | **1** |
| --- | --- | --- | --- | --- | --- | --- | --- | --- | --- |

3- How confident are you that you have the necessary skills to replace waterpipe smoking with other healthy recreations?

| **10** | **9** | **8** | **7** | **6** | **5** | **4** | **3** | **2** | **1** |
| --- | --- | --- | --- | --- | --- | --- | --- | --- | --- |

1. How confident are you that you can resist the compliments of people around you to smoke waterpipe?

| **10** | **9** | **8** | **7** | **6** | **5** | **4** | **3** | **2** | **1** |
| --- | --- | --- | --- | --- | --- | --- | --- | --- | --- |

1. How confident are you that you can use effective methods in the tempting conditions of waterpipe smoking?

| **10** | **9** | **8** | **7** | **6** | **5** | **4** | **3** | **2** | **1** |
| --- | --- | --- | --- | --- | --- | --- | --- | --- | --- |

6- How confident are you that you can tolerate the side effects and symptoms of waterpipe cessation?

| **10** | **9** | **8** | **7** | **6** | **5** | **4** | **3** | **2** | **1** |
| --- | --- | --- | --- | --- | --- | --- | --- | --- | --- |

| **10** | **9** | **8** | **7** | **6** | **5** | **4** | **3** | **2** | **1** |
| --- | --- | --- | --- | --- | --- | --- | --- | --- | --- |

7. How confident are you that you can break up with waterpipe smoking friends?

1. How confident are you that success in quitting waterpipe is due to your inner strength and will?

| **10** | **9** | **8** | **7** | **6** | **5** | **4** | **3** | **2** | **1** |
| --- | --- | --- | --- | --- | --- | --- | --- | --- | --- |

**9**- How confident are you that despite the habit of smoking waterpipe, you will quit it?

| **10** | **9** | **8** | **7** | **6** | **5** | **4** | **3** | **2** | **1** |
| --- | --- | --- | --- | --- | --- | --- | --- | --- | --- |
